# The Potential of Artificial Intelligence in Pharmaceutical Innovation: From Drug Discovery to Clinical Trials

**DOI:** 10.3390/ph18060788

**Published:** 2025-05-25

**Authors:** Vera Malheiro, Beatriz Santos, Ana Figueiras, Filipa Mascarenhas-Melo

**Affiliations:** 1 Laboratory of Drug Development and Technologies, Faculty of Pharmacy, University of Coimbra, 3000-548 Coimbra, Portugal; veracatarina.am13@gmail.com (V.M.); beatrizcsantos.trabalho@gmail.com (B.S.); 2 REQUIMTE/LAQV, Group of Pharmaceutical Technology, University of Coimbra, 3000-548 Coimbra, Portugal; 3 Higher School of Health, Polytechnic Institute of Guarda, Rua da Cadeia, 6300-307 Guarda, Portugal; 4 BRIDGES—Biotechnology Research, Innovation and Design for Health Products, Polytechnic University of Guarda, Avenida Dr. Francisco Sá Carneiro, n.° 50, 6300-559 Guarda, Portugal

**Keywords:** artificial intelligence, machine and deep learning, drug discovery and development, drug repurposing, clinical trials, regulatory issues

## Abstract

Artificial intelligence (AI) is a subfield of computer science focused on developing systems that can execute tasks traditionally associated with human intelligence. AI systems work through algorithms based on rules or instructions that enable the machine to make decisions. With the advancement of science, more sophisticated AI techniques, such as machine learning and deep learning, have been developed, allowing machines to learn from large amounts of data and improve their performance over time. The pharmaceutical industry has greatly benefited from the development of this technology. AI has revolutionized drug discovery and development by enabling rapid and effective analysis of vast volumes of biological and chemical data during the identification of new therapeutic compounds. The algorithms developed can predict the efficacy, toxicity, and possible adverse effects of new drugs, optimize the steps involved in clinical trials, reduce associated time and costs, and facilitate the implementation of innovative drugs in the market, making it easier to develop precise therapies tailored to the individual genetic profile of patients. Despite significant advancements, there are still gaps in the application of AI, particularly due to the lack of comprehensive regulation. The constant evolution of this technology requires ongoing and in-depth legislative oversight to ensure its use remains safe, ethical, and free from bias. This review explores the role of AI in drug development, assessing its potential to enhance formulation, accelerate discovery, and repurpose existing medications. It highlights AI’s impact across all stages, from initial research to clinical trials, emphasizing its ability to optimize processes, drive innovation, and improve therapeutic outcomes.

## 1. Introduction

The development of medicines has always been a long, costly process with a low success rate [1]. In recent years, there has been significant progress in the pharmaceutical industry, requiring the adoption of more efficient and systematic drug production practices [1]. Drug discovery is driven by the fact that there are no treatments available for some diseases, or existing drugs have low efficacy or high toxicity [1]. To address these challenges, advancements in technology have provided a wide range of techniques widely used to accelerate all stages of drug development, which have been radically transformed by the rapid progress of AI [2].

AI was described in the 1950s as a science and engineering field capable of developing intelligent machines. It quickly evolved into a neural network model similar to the human brain, enabling the execution of tasks requiring human intelligence, such as solving complex problems and making complex decisions. AI has evolved exponentially and is becoming a highly important and revolutionary component in many areas, particularly in drug discovery [2,3].

The exponential growth of AI has raised several ethical issues. The ability of these technologies to make autonomous decisions raises concerns about human dignity and inherent values. For this reason, it has become necessary to establish ethical guidelines to prevent significant consequences for human life. Ethics is a practical necessity to ensure that technologies are used in a fair and responsible way [4].

In the drug development process, AI is not limited to discovering new drugs. It is also characterized by the optimization of existing treatments tailored to the individual profile of each patient. This approach improves treatment efficacy, minimizes side effects, and provides safer and more effective therapeutic solutions [5,6].

In this context, the main objective of this review is to evaluate the potential of AI in drug formulation and how this technology can effectively contribute to the discovery and development of new medications, as well as the adaptation of existing medications to other diseases, thus providing a safer and more effective therapeutic experience.

## 2. Artificial Intelligence

Artificial intelligence (AI) is increasingly integrated into pharmaceutical research, revolutionizing processes across drug discovery, development, and clinical decision-making. AI encompasses technologies that simulate human intelligence to perform tasks such as learning, reasoning, and pattern recognition [7]. AI is an interdisciplinary field combining computer science, statistics, and domain-specific knowledge. It is often described as a set of technologies, processes, and methods that enable machines to perform cognitive functions, such as reasoning and decision-making [8]. In the context of pharmaceutical research, AI models can accelerate compound screening, predict molecular interactions, and optimize clinical trial designs. While traditional definitions of AI cover a wide scope, from narrow to general intelligence, this work emphasizes those tools and frameworks with demonstrated relevance to biomedical and pharmaceutical applications [9].

**Figure 1:**
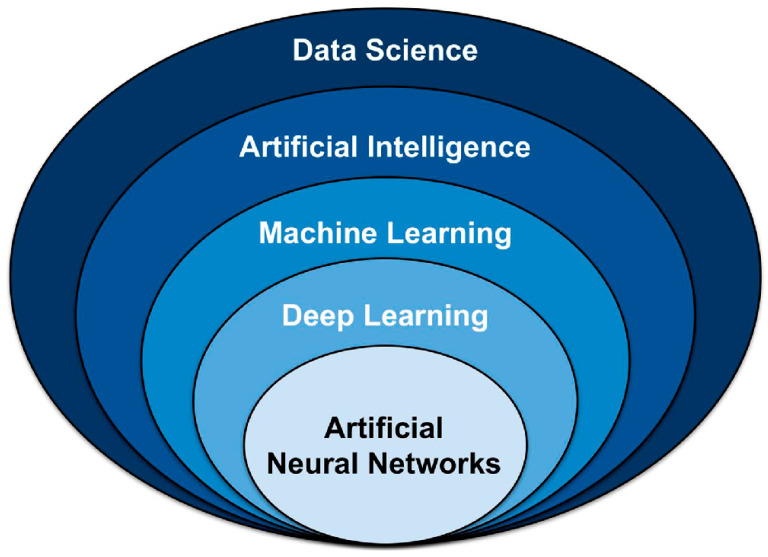
Overview of key data science techniques. AI (AI) is a subset of data science that encompasses both classical programming and machine learning (ML). Within ML, various models and approaches exist, including deep learning (DL) and artificial neural networks (ANNs). Adapted from [14].

Talking about AI inevitably involves referencing the concepts of machine learning (ML) and deep learning (DL). While these concepts are related, they are not interchangeable. AI focuses on intellectual activities typically performed by humans, such as solving well-defined logical problems, whereas ML and DL are specific methods that help achieve this, such as voice recognition or image classification [10]. In other words, both ML and DL operate within the broader scope of AI (Figure 1).

### 2.1. From the Beginning of AI to the Present Day

Charles Babbage became one of the leading mathematicians of his time and is often referred to as the “father of the computer” for creating the first modern analytical machine, which he described as the first thinking machine. Later, he conceived the analytical machine with the intention of developing precise mathematical tables [7]. Subsequently, Alan Turing was recognized as the “father of computation and AI” when, in 1950, he published the article *Computers and Intelligence*, in which he described the ability of computers to exhibit intelligence similar to human intelligence, an idea that became known as the “Turing Test” [3]. This test is based on an “imitation game”, which assesses a person’s ability to distinguish between a conversation with a human and one with a machine [11]. Following Turing, the field became more complex, requiring the exploration of new challenges, which contributed to advancements in computer science and mathematics. This journey leading to the modern AI we know today is concisely outlined in Table 1.

**Table 1:** AI progress: major breakthroughs over time.

Year	Progress Information	References
1955	Allen Newell, J.C. Shaw, and Herbert A. Simon created a program called the “Logic Theorist”, the first program capable of performing automated reasoning—the first AI software in operation. In the following year, the program was demonstrated for the first time in Pittsburgh.	[7]
1956	John McCarthy first introduced the term AI at a conference on the subject. Later, in 1958, McCarthy developed the programming language *Lisp*, which became fundamental to the advancement of computer science and AI. Alongside Marvin Minsky, in 1959, he founded the AI laboratories.	[7,12]
1958	Arthur Samuel coined the term “Machine Learning” when he wrote about teaching a machine to play chess better than the humans who created it.	[7]
1965	Edward Feigenbaum initiated *DENDRAL*, the first “expert system”, capable of extracting molecular structures from organic compounds using scientific data.	[7]
1966	Joseph Weizenbaum developed the first “chatterbot”, *ELIZA*, a computer program designed to simulate a fictional psychotherapist. ELIZA used natural language processing to communicate with humans.	[7]
1979	The first American Association for AI was founded, currently known as the Association for the Advancement of AI (AAAI).	[7]
1980s	The beginning of this decade became known as the “AI Boom”, as rapid growth and interest in AI emerged. John Hopfield and David Rumelhart introduced deep learning techniques, enabling computers to learn from experiences. Edward Feigenbaum developed specialized systems to imitate human decision-making processes. These systems were widely adopted in industry. The year 1984 became known as the “AI winter” because consumer, public, and private sectors began to show little interest in AI, leading to a decline in funding and research.	[7]
1995	Stuart Russell and Peter Norvig published *“AI: A Modern Approach”*, which became one of the key textbooks in the field. Richard Wallace developed the chatbot *ALICE*, which established more natural conversations with users.	[7]
1997	IBM’s specialized chess-playing system, *Deep Blue*, defeated Garry Kasparov, the reigning world chess champion and grandmaster, in a historic chess match. Dragon Systems developed voice recognition software, which was implemented in Windows.	[7,13]
2000	Cynthia Breazeal developed *Kismet*, the first robot capable of simulating human emotions, mimicking human facial expressions.	[7]
2002	iRobot introduced the Roomba, the first robotic vacuum cleaner that revolutionized the way homes were cleaned. Equipped with intelligent sensors, the Roomba navigates autonomously, detecting and avoiding obstacles, making cleaning easier and more efficient.	[7]
2010	Microsoft introduced Kinect for the Xbox 360, the first gaming device capable of tracking body movements and converting them into commands within the game.	[7]
2011	Apple launched Siri, the first virtual assistant.	[7]
2014	Amazon created the virtual assistant Alexa, allowing users to interact with electronic devices using voice commands.	[7]
2016	Hanson Robotics developed Sophia, the first humanoid robot with a realistic appearance, capable of communicating, seeing, and replicating human emotions.	[7]
2020	DeepMind released an updated version of AlphaFold, a program capable of deciphering the three-dimensional structure of any protein.	[7]
2022	OpenAI launched the chatbot *ChatGPT*	[7]

### 2.2. Machine Learning

Machine learning is recognized as a subset of AI, involving algorithms with the ability to define their own rules based on input data without explicit programming. It primarily uses two methods for data analysis [10]. In the supervised method, the system is programmed to recognize specific patterns previously discovered, meaning the system has access to the desired responses, facilitating the process of classification and grouping ideas. On the other hand, the unsupervised method detects an unknown set of data and categorizes it into specific combinations independently [11].

It can also be divided into another method, known as reinforcement learning. This is a trial-and-error approach, driven mainly by decision-making within a specific environment and the execution of actions to maximize performance. The goal of this method is to guide situations through its actions [12,14].

#### Common AI Models in Pharmaceutical Applications

Recent years have seen a marked increase in the use of machine learning models to tackle complex problems in drug development, toxicology, and personalized medicine. Among the machine learning techniques applied in the pharmaceutical field, several models stand out due to their versatility and performance. One such model is random forest, an ensemble method that builds multiple decision trees and combines their outputs to improve prediction accuracy and minimize overfitting. This approach has proven effective in tasks such as classifying toxicity profiles and identifying potential biomarkers in preclinical research [15].

Another widely adopted model is the artificial neural network (ANN), inspired by the structure of the human brain. ANNs consist of layers of interconnected nodes (or “neurons”) capable of recognizing complex patterns in large datasets. In pharmaceutical research, they have been successfully used in areas ranging from molecular property prediction to clinical decision support and image-based drug screening [16]. Recent advances in generative models, particularly Generative Adversarial Networks (GANs), have introduced new possibilities in drug design. By leveraging a dual-network system—comprising a generator and a discriminator—GANs are able to produce novel molecular structures that meet predefined pharmacological criteria, thus accelerating early-stage drug discovery [17,18].

In parallel, Transformer architectures, originally developed for natural language processing, are now being repurposed to handle biomedical data. Their self-attention mechanism allows them to process large-scale biological sequences, predict drug–target interactions, and extract insights from unstructured clinical data with remarkable precision. These models are increasingly being integrated into platforms aimed at personalized medicine and advanced therapeutic development [19].

### 2.3. Deep Learning

Deep learning is seen as an extension of the ML method, as while the latter relies on more traditional approaches to reach its answers, DL is more complex and consists of a set of algorithms aimed at mimicking the capabilities of the human brain [11,13]. It builds on the evolution of the artificial neural network (ANN)—a set of multilayered units of nonlinear processing used for data representation, which includes sophisticated computational elements connected to one another, resembling “perceptions” comparable to human neurons. These systems replicate the transmission of electrical signals observed in the human brain [11]. These models are particularly effective in image analysis (e.g., histopathology, radiology), drug–target interaction prediction, and modeling protein structures.

### 2.4. Ethical Concerns in the Use of AI in Healthcare

The integration of artificial intelligence (AI) into pharmaceutical research and healthcare systems introduces significant ethical considerations, especially in areas such as data privacy, algorithmic bias, transparency, and human oversight. AI technologies are capable of analyzing and processing vast amounts of data, supporting decisions in clinical trial design, patient stratification, and the prediction of drug response [4,9,20]. While these capabilities promise major advances, they also raise questions about how to ensure the fair and responsible use of AI.

Ethical principles, understood as duties that promote human dignity in the presence of intelligent systems, must guide the development and deployment of AI in drug development contexts. Although these principles are universal in nature, their application can vary across different cultural, legal, and social settings [14]. As healthcare becomes more data driven and patient centered, AI is increasingly seen as a valuable support tool. However, maintaining human control over clinical decisions remains essential. Human dignity and fundamental values—such as the obligation to avoid harm, ensure fairness, and prevent discrimination—must remain central to the use of AI [4]. This is particularly crucial when AI is involved in sensitive areas like patient selection for trials or prediction of adverse events, where biases in training data can exacerbate existing health inequities.

To ensure ethical integrity, values such as accountability, transparency, explainability, and privacy should be embedded into AI systems from the outset [4]. Table 2 outlines widely accepted ethical principles designed to complement existing human rights standards and guide responsible AI deployment in healthcare.

Regulatory bodies, such as the European Medicines Agency (EMA) and the U.S. Food and Drug Administration (FDA), are actively working to establish frameworks that assess both the technical and ethical robustness of AI-based tools. For instance, the EMA’s 2023 Reflection Paper on Artificial Intelligence emphasizes the importance of ethical oversight across the entire AI lifecycle, from data collection to real-world deployment.

Ultimately, ethical governance must involve a multi-stakeholder approach, including developers, clinicians, industry, and regulators, ensuring that AI technologies serve the collective good while safeguarding individual rights.

## 3. Challenges and Transformations in R&D Through AI Integration

Pharmaceutical companies face significant challenges in the development of new medications, primarily due to the extremely high costs and low success rates associated with the process. Since 2001, drug development expenses have risen by several billion dollars, making cost the leading factor behind inefficiencies in research and development [22,23]. Despite these investments, the return remains low, largely because of high failure rates—especially regarding toxicity and efficacy.

The clinical trial success rate is under 15%, and approximately half of drug discovery failures are due to poor pharmacokinetic properties in the preclinical phase. Although Phase I trials usually confirm safety, many candidates fail in Phase II because of insufficient efficacy. Additionally, population heterogeneity contributes to this lack of success [22,23].

Traditionally, drug discovery has followed a linear and highly regulated pathway, from target identification through preclinical testing to clinical trials, often taking 10 to 15 years and billions of dollars to bring a single drug to market [24]. This approach was historically centered on single-target drug development, but modern strategies have since evolved to enhance efficiency [24,25]. Consequently, there is a growing need to adopt innovative technologies that can improve the overall R&D process.

**Figure 2:**
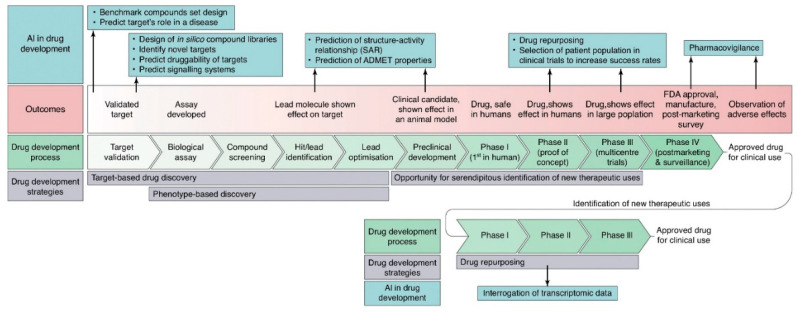
Schematic representation of the application of AI in each stage of the drug development process. Adapted with permission from Ref. [12]. Copyright 2019 Elsevier.

The integration of AI (AI) into drug development is explored in Figure 2, detailing the strategies and outcomes of its key components. Additionally, AI applications at each stage of the process are highlighted.

These paradigm shifts in drug development, especially the move toward data-intensive approaches, such as high-throughput screening and drug repurposing, have set the stage for the integration of artificial intelligence. The following sections explore how AI is being applied across these evolving domains, from molecule design to clinical trial execution.

### 3.1. Application of AI in Formulation

AI has begun to drive its application across various sectors of society, with the pharmaceutical industry emerging as a primary beneficiary. Advancements in AI have shown the potential to discover and develop medications more quickly and at a lower cost [5]. Over the years, there has been a dramatic increase in data input within the pharmaceutical sector, motivating the use of AI to automate and optimize slow processes, thereby significantly accelerating the research and development of medications due to its capability to manage large volumes of data [5].

When applied in the formulation of pharmaceutical products, AI can be utilized at various stages of drug development, including the identification and validation of potential targets, drug design and screening, drug repositioning, improving R&D efficacy, analyzing biomedical information, quality control, pharmaceutical product management, and assisting in the selection of patients for clinical trials [5,12]. The search for new drugs that will be successful in the future is perhaps the most challenging part of the drug development process, as the estimated number of viable molecules with potential interest in developing new medications ranges between 10^60^ and 10^100^ [12,26]. A viable molecule is defined as one that meets Lipinski’s rule of five, which is a computational procedure used to estimate the solubility, permeability, and pharmacological efficacy in drug discovery. According to this rule, molecules must have a molecular weight of less than 500 Da, a number of hydrogen bond donors of less than 5, a number of hydrogen bond acceptors of less than 10, and a LogP of less than 5 [26]. However, the lack of advanced technology limits the process of selecting viable molecules and, consequently, the development of medications, making this process slow and costly, which could be resolved through the use of AI [26].

The virtual chemical space is vast and provides a geographical map of molecules, illustrating their properties and interactions between molecules, facilitating the synthesis of information about compounds that could produce desired therapeutic effects, and also selecting molecules with properties suitable for further testing [5,27]. There are several databases available, such as PubChem, which is freely accessible and provides information on chemical molecules and their activities in biological assays, and ZINC, which contains a vast number of chemical compounds and is designed to facilitate virtual screening. It is essentially organized to assist techniques like molecular docking—virtual simulations of interactions between molecules and protein binding sites, helping to identify new molecules with pharmacological activity [27].

The data available in pharmaceutical companies for developing medications can involve various compounds, and ML tools may struggle to handle this type of data. For this reason, a computational model called QSAR (Quantitative Structure–Activity Relationship) was developed, which can quickly predict a large number of compounds and their physicochemical parameters [5,28]. However, this model cannot predict complex biological properties, such as efficacy and potential adverse effects of compounds. Moreover, QSAR models encounter several challenges, including limited training datasets, inaccuracies in the experimental data, and insufficient experimental validation. To address these limitations, more sophisticated AI methodologies, such as deep learning combined with relevant modeling studies, have been incorporated into the evaluation of drug safety and efficacy through the application of big data analytics [5,28].

#### 3.1.1. Drug Screening

In the preclinical phase, high-throughput screening, omics technologies, and computational tools are now routinely used to evaluate thousands of compounds in parallel. These approaches allow for the simultaneous testing of multiple targets, greatly expanding the number of viable candidates and accelerating early discovery stages. The inclusion of AI in drug discovery has proven to be more effective than in vitro screening. Virtual screening is the most commonly used method to aid in the discovery of bioactive molecules, selecting a viable compound from the vast pool available in databases. Therefore, drug screening involves two main components: the preparation of the compound library and the selection of desirable compounds [29,30].

The SMACC (Small Molecule Antiviral Compound Collection) is a highly curated and meticulously annotated library of compounds tested in phenotypic assays and antiviral-based screening for viruses [29].

Moreover, AI plays a crucial role in the screening process, as it can predict toxicity, bioactivity, ADME properties, and the physicochemical properties of molecules under investigation [30].

**Figure 3:**
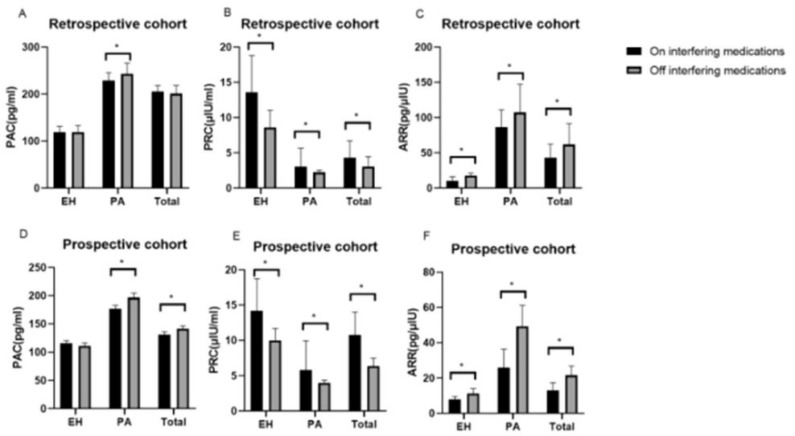
Variations in plasma aldosterone concentration (PAC), plasma renin concentration (PRC), and the aldosterone-to-renin ratio (ARR) observed in patients with essential hypertension (EH) and primary aldosteronism (PA) before and after the discontinuation of interfering antihypertensive medications. (**A**) PAC levels in the retrospective cohort, on and off interfering medications; (**B**) PRC levels in the retrospective cohort, on and off interfering medications; (**C**) ARR values in the retrospective cohort, on and off interfering medications; (**D**) PAC levels in the prospective cohort, on and off interfering medications; (**E**) PRC levels in the prospective cohort, on and off interfering medications; (**F**) ARR values in the prospective cohort, on and off interfering medications. * Statistically significant difference (*p* < 0.05). Reprinted with permission from Ref. [32]. Copyright 2023 Springer Nature.

**Figure 4:**
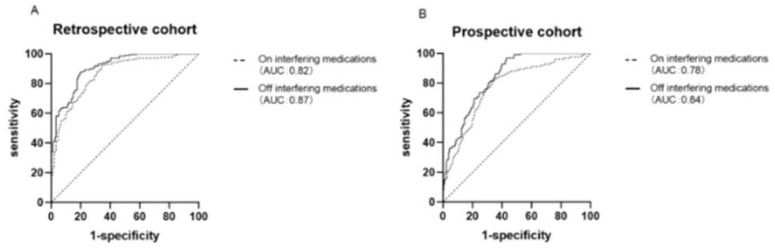
Receiver operating characteristic (ROC) curves comparing the diagnostic performance of the aldosterone-to-renin ratio (ARR) with and without interfering with antihypertensive medications in retrospective (**A**) and prospective (**B**) cohorts. The analysis illustrates how data-driven threshold optimization—analogous to AI-driven calibration—can maintain diagnostic accuracy despite clinical variability. Reprinted with permission from Ref. [32]. Copyright 2023 Springer Nature.

One particularly challenging domain where AI has shown promise is the prediction of interactions between conventional drugs and herbal medicinal products, which are increasingly used as dietary supplements. These natural products typically consist of complex mixtures with poorly characterized pharmacokinetics, making traditional interaction screening methods less effective. AI algorithms can integrate large-scale data, including chemical structures, molecular pathways, pharmacological properties, and known interaction patterns, to infer potential risks and mechanistic pathways of drug–herb interactions. A recent review highlighted how AI tools can aid in identifying high-risk combinations, offering valuable support for pharmacovigilance and experimental validation. As the use of herbal supplements grows globally, the incorporation of AI in this context represents a crucial advance in drug safety assessment [31]. AI-driven methods have proven particularly useful in optimizing diagnostic parameters, including thresholds for biomarker-based screening tools. A recent study investigating the aldosterone-to-renin ratio (ARR) for screening primary aldosteronism (PA) demonstrated that with appropriate adjustment of the ARR threshold, screening remains accurate even in patients taking interfering antihypertensive medications. As illustrated in Figure 3, the pharmacological context significantly alters the underlying biomarker values (PAC, PRC, and ARR), necessitating adaptive analytical frameworks. Through receiver operating characteristic (ROC) curve analysis (Figure 4), the authors showed that lowering the ARR threshold in treated patients preserved sensitivity and specificity comparable to standard protocols. These results illustrate the value of data-centric, adaptive approaches, often implemented using AI, in refining clinical decision-making and ensuring diagnostic robustness across real-world patient conditions [32].

While small molecules have traditionally dominated early-stage drug discovery efforts, particularly due to their favorable physicochemical properties and oral bioavailability, recent advances in AI have increasingly been extended to the design and optimization of biotechnological products, including monoclonal antibodies, therapeutic peptides, and nucleic acid-based therapies. These complex molecules pose unique challenges, such as stability, immunogenicity, and production scalability, which are now being addressed through machine learning algorithms trained on structural, genomic, and pharmacokinetic datasets [33,34,35].

In this context, traditional heuristics, such as Lipinski’s Rule of Five, remain useful for the initial filtering of small molecules but have limited applicability in the design of larger or more complex entities. Moreover, AI-driven discovery platforms are beginning to surpass these empirical rules by identifying candidates that fall outside conventional boundaries yet still demonstrate clinical efficacy. For example, deep learning models that consider 3D conformations, protein–ligand interactions, and dynamic behavior are revealing new “druggable” spaces [36].

The integration of tools like AlphaFold, which accurately predicts protein structures, and the rise of explainable AI (XAI) approaches, which provide interpretability and mechanistic insights, further indicate a shift from traditional rule-based paradigms toward data-driven and structure-informed discovery frameworks. These innovations are expected to expand the range of viable therapeutic candidates and improve the rational design of both small and large molecules [36].

##### Toxicity Prediction

The toxicity of a drug is a crucial parameter in the drug development process, referring to adverse effects caused by the drug’s mechanism of action or metabolites in the body [28,30]. Toxicity tests are initially conducted in vitro on cells, followed by studies in animals to identify the compound’s toxicity in a living organism. These methods increase the cost and extend the time required for drug development [37].

Several tools and pipelines are used to predict the toxicity of compounds. For instance, eToxPred predicts bioavailability and toxicity through molecular fingerprints [30]. The Tox21 Data Challenge is a competition where researchers tested thousands of environmental chemicals and drugs for twelve different toxic effects. This led to the creation of the DeepTox algorithm, which first normalizes the chemical representations of the products and calculates numerous molecular properties used as input for machine learning methods. Then, these chemical properties are grouped, and DeepTox predicts the toxicity of new compounds [37].

##### Bioactivity Prediction

The effectiveness of a drug hinges on its binding affinity to the target protein or receptor. Molecules that fail to interact or attach to the target protein are unlikely to trigger a therapeutic response [5]. Sometimes, the drugs being studied may interact with unintended proteins or receptors, leading to toxicity. Therefore, it is essential to understand the drug’s affinity for its receptor (DTBA—Drug–Target Binding Affinity) in order to predict drug–receptor interactions. Algorithms like ChemMapper are designed to predict such interactions [5,30]. The contribution of AI in this field has been demonstrated through advancements in the analysis of molecular pairs (MMP—Matched Molecular Pair), where the impact of a single molecular change on molecular properties and bioactivity is investigated. This approach is widely used in QSAR testing [28]. A candidate molecule is chemically defined by a static core and two fragments that are subsequently encoded. Machine learning methods, applied after the MMP, can be used again to extrapolate possible new transformations, fragments, and modifications of the static core [28]. DL approaches outperform ML methods in some cases since they do not rely on the availability of 3D protein structures. Examples of such DL methods include DeepDTA, which predicts and analyzes interactions between drugs and target proteins, and DeepAffinity, which considers amino acid sequences and molecules for structural and physicochemical properties, among others [5,30].

##### Prediction of Physicochemical Properties

Physicochemical properties—solubility, logP (partition coefficient), ionization degree, intrinsic permeability, lipophilicity, and melting point—must be considered when designing a new medication, as they can impact pharmacokinetic properties and interaction with receptors [5,37]. LogP, which is the ratio of a substance’s concentration between an organic solvent and water, helps estimate the drug’s cellular absorption. On the other hand, the melting point indicates the ease with which a drug dissolves in a medium [37].

Considering the above properties, molecular representations used in AI algorithms for drug design include “fingerprint”, a method that represents the molecule as a sequence of numbers, as well as a simplified molecular input line entry system (SMILES) for predicting molecular properties, molecular graphs where atoms are represented as points and bonds as edges, and others [38]. Applying algorithms facilitates the prediction of molecular properties and allows for the identification of potential relationships between molecular properties and their structures. For example, the EPI (Estimation Program Interface) program allows for the evaluation of certain physicochemical properties through the application of the QSPR (Quantitative Structure–Property Relationship) algorithm [39].

#### 3.1.2. Drug Design

The vast availability of data presents a significant challenge for drug design, as it involves navigating an expansive chemical information space associated with various targets to identify the optimal solution [40]. Several milestones have been achieved in the drug design process, establishing it as the primary approach for the present and future. The first major advancement was recognizing and understanding drug–receptor interactions. Daniel Koshland demonstrated that both molecules undergo conformational changes during their interaction, adopting the most suitable conformations to bind effectively [41]. With technological advancements and the integration of AI, the primary breakthrough in this field has been the development of in silico modeling technologies applied to virtual screening, compound design, SAR and QSAR analyses, pharmacokinetic modeling, and drug–drug interaction studies [41]. AI plays a critical role in drug design by anticipating protein–drug interactions and predicting the structure of target proteins [39].

##### Prediction of Drug–Protein Interaction

For a drug to have a therapeutic effect, it must first bind to a protein to ensure effective treatment of a disease. Understanding drug–protein interactions is crucial for optimizing drug efficacy and effectiveness. This knowledge is instrumental in avoiding unintended drug repurposing and mitigating polypharmacology-related issues [5]. These interactions are fundamental and are often directly linked to various diseases through distinct biological processes. They involve numerous protein binding sites beyond the conventional targets, such as ion channels, kinases, and nuclear receptors, facilitating the development of small-molecule drugs. However, existing AI methods in this area face limitations due to the availability and diversity of structural data, as only a small fraction of known structures include detailed insights into yeasts, microorganisms, and human proteins [42].

##### Prediction of Target Protein 3D Structure

When the goal is to develop a molecule that will later be integrated into a medication targeting a specific disease, predicting the structure of the target protein is crucial for ensuring a successful therapeutic outcome [5]. The 3D structure of the target protein is critical because new drug molecules are designed based on the chemical environment of the target protein’s binding site [37]. However, predicting the three-dimensional structure of a protein is challenging, as it relies solely on its amino acid sequence, making this task highly complex [43].

Advancements in technology have led to the development of useful methods and tools to assist researchers in addressing this issue. Among these is AlphaFold, the first computational method capable of predicting protein structures with atomic precision, even when no similar structure is known [37,43]. AlphaFold utilizes deep neural networks (DNNs) to predict protein properties from its primary amino acid sequence. It analyzes the distance between adjacent amino acid pairs and the corresponding peptide bond angles to accurately predict the protein’s three-dimensional structure. AlphaFold successfully predicted 25 out of 43 protein structures with remarkable precision, surpassing other methods by a significant margin [37,43,44].

#### 3.1.3. Drug Repurposing

In parallel to the new drug development, drug repurposing, the identification of new therapeutic uses for existing or previously shelved drugs, has become an increasingly strategic and cost-effective alternative. This approach circumvents early preclinical testing and leverages existing safety and pharmacokinetic data to advance candidates more rapidly into clinical evaluation. When combined with AI technologies, drug repurposing becomes even more powerful, as machine learning models can mine large-scale biomedical datasets, molecular structures, and disease networks to identify novel indications [45].

**Figure 5:**
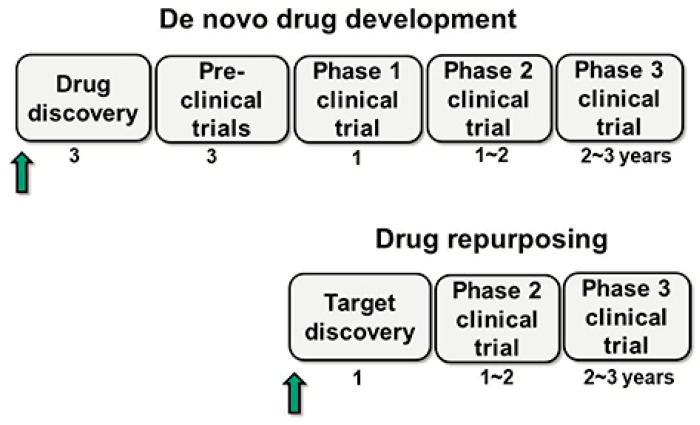
Schematic representation of the new drug development and drug repurposing. The arrow indicates the starting point for each approach, while the numbers represent the duration in years. In this figure, drug repurposing is considered the process of identifying or discovering new therapeutic targets for an already marketed drug. Unlike the new development, repurposing begins with target discovery and proceeds directly to Phase 2 and 3 clinical trials, bypassing animal studies and Phase 1 trials, as existing data from the original drug can be leveraged. Adapted from [46].

Drug repurposing involves discovering new therapeutic uses for already approved drugs (Table 3) [45]. The main advantages of this approach include pre-existing knowledge of toxicity, efficacy, and pharmacokinetic and pharmacodynamic profiles. This allows the drug to proceed directly to Phase 2 or Phase 3 clinical trials, significantly reducing associated costs and development time (Figure 5) [6,46].

Drug repurposing can be carried out experimentally or through computers, with the latter method being referred to as in silico drug repurposing, which can be classified according to two approaches: discovering new indications for an existing drug—drug centered—or identifying effective drugs for diseases—disease centered [46].

The concept of in silico drug repurposing is currently widely used, and its expansion was made possible due to two types of technologies, such as the high-throughput data collection from various sources, including genomics, proteomics, chemoproteomics, and phenomics, which were generated and stored, and due to advances in computational and data sciences that enabled the development of repositioning algorithms along with retrospective analyses and maintenance of experimental data in a database [46].

This strategy includes **target-based drug repurposing,** which screens almost all drug compounds with known chemical structures and directly links targets to disease mechanisms; **pathway-based repurposing**, which utilizes metabolic and signaling pathways and information about protein interactions to predict the similarity or connection between drugs and diseases; and **mechanism-based repurposing**, which interlinks information about signaling pathways, treatment-related omics data, and protein interaction networks to discover new mechanisms of action for drugs [46].

ML techniques applied to drug repurposing include logistic regression, support vector machine (SVM), random forest, neural forest (NN), and DL [46].

Ormeloxifene, originally developed as a non-steroidal oral contraceptive, has been repurposed for cancer treatment using AI-driven approaches. Machine learning algorithms analyzing gene expression data and molecular docking studies have identified its potential in targeting signaling pathways involved in cancer progression, particularly in breast and ovarian cancers. By leveraging deep learning models trained on drug–target interaction networks, researchers have found that ormeloxifene exhibits anti-cancer properties by modulating cell cycle regulation and apoptosis [47].

Widely used for type 2 diabetes, metformin has gained attention as a potential anti-cancer agent through deep learning models that analyze large-scale patient datasets, gene expression profiles, and cancer cell line responses. AI-based techniques, particularly unsupervised clustering and neural networks, have linked metformin to the inhibition of the mTOR and AMPK pathways—key regulators of cancer metabolism and proliferation. These findings have led to multiple clinical trials investigating metformin’s role in preventing and treating various cancers, including breast and colorectal cancer [47].

Initially developed as an H2 receptor antagonist for gastric ulcers and acid reflux, cimetidine was identified as a potential cancer therapy through AI-based drug repurposing pipelines. Computational methods, including network-based drug discovery and virtual screening, have demonstrated that cimetidine can modulate immune response and inhibit cancer cell adhesion, particularly in colorectal and gastric cancers. AI-driven molecular docking studies suggest that cimetidine disrupts tumor immune evasion by blocking E-selectin-mediated cancer metastasis, making it a promising candidate for combination cancer therapies [47].

Finally, originally approved for osteoporosis treatment, bazedoxifene has been repurposed for oncology using deep learning techniques that analyze molecular binding affinities and structural similarities with known anti-cancer agents. AI-driven screening of protein–ligand interactions has shown that bazedoxifene acts as a potent STAT3 inhibitor, a key target in various cancers, including breast and pancreatic cancers. By leveraging reinforcement learning and generative models, researchers have further optimized its anti-cancer potential, leading to preclinical studies exploring its role in targeted cancer therapies [47].

These examples highlight how AI techniques—ranging from machine learning on biological data to deep learning-based molecular simulations—are accelerating drug repurposing efforts, offering new therapeutic possibilities for cancer treatment [47].

#### 3.1.4. Polypharmacology

The concept of polypharmacology shifts from the traditional “one drug, one target” approach and explores how different drugs can interact with multiple pharmacological targets, potentially producing side effects or therapeutic benefits [48,49]. Polypharmacology involves addressing several challenging issues in drug discovery, including identifying synergistic targets, designing chemical molecules with the desired multi-target profile, and evaluating their bioactivity [48,49]. The emergence of the concept of multifactorial diseases required the involvement of multi-target (MT) approaches, becoming a key focus in various therapeutic areas. Drug interactions with different targets could lead to unexpected side effects, involving “anti-targets”, such as ion channels or metabolizing enzymes. However, not all these unintended effects are undesirable, as they provide a basis for drug repurposing [48].

AI has helped revolutionize this concept of polypharmacology by providing extensive knowledge about drugs and their interactions. SynPhayhy, an ML-based approach, uses a neural network based on drug chemical structures, offers insights into drug synergy, and identifies relevant biological pathways. DeepMDS also predicts synergy between multiple drugs based on therapeutic targets and gene expression information [49].

#### 3.1.5. Integration of AI with PBPK and Population PK Models

The integration of artificial intelligence with Model-Informed Drug Development (MIDD) offers a powerful paradigm shift in pharmaceutical R&D. MIDD approaches, such as physiologically based pharmacokinetic (PBPK) and population pharmacokinetic (Pop-PK) modeling, rely on mechanistic simulations to predict drug behavior under various physiological conditions. When enhanced by AI techniques, including machine learning, deep learning, and generative algorithms, these models can incorporate complex, real-world datasets to refine predictions, simulate virtual populations, and optimize dosing strategies. This synergistic integration allows for more accurate hypothesis generation, derisking of candidates, and acceleration of development timelines, particularly in early-phase trials. However, successful implementation depends on the availability of high-quality data, robust validation processes, and attention to interpretability and algorithmic fairness. Regulatory agencies have begun adapting their evaluation frameworks to accommodate this hybrid approach, acknowledging its transformative potential for drug discovery and personalized medicine [50].

Pharmacokinetic modeling has long been essential for understanding drug absorption, distribution, metabolism, and excretion (ADME) characteristics. Among the most widely used approaches are physiologically based pharmacokinetic (PBPK) models and population pharmacokinetic (Pop-PK) models. PBPK models simulate drug behavior using mathematical representations of physiological systems, incorporating variables such as tissue volumes, blood flow, and enzyme activity. Pop-PK models, by contrast, analyze variability in drug kinetics across diverse patient populations to support dose optimization and safety assessments [51,52]. The integration of AI and machine learning techniques into PBPK and Pop-PK modeling has significantly enhanced predictive accuracy and efficiency. AI algorithms can process vast clinical and preclinical datasets to automatically calibrate model parameters, identify nonlinear patterns, and generate simulations that would be unfeasible using conventional methods alone. This hybrid modeling approach allows for more accurate dose prediction, better inter-subject variability characterization, and faster regulatory submissions [53,54]. Moreover, AI-enhanced PBPK models have been used to anticipate drug–drug interactions, simulate scenarios in pediatric or geriatric populations, and guide first-in-human (FIH) dose selection. In combination with real-world data and omics-based biomarkers, these models are also instrumental in precision dosing strategies. By reducing the number of empirical studies required and enabling earlier go/no-go decisions, the fusion of AI with pharmacokinetic modeling contributes directly to cost reduction, faster development, and improved clinical outcomes [55,56].

### 3.2. Integrating AI in Clinical Trials

The data generated from clinical trials form the basis for evidence-based medicine, guiding healthcare professionals in making informed decisions about patient care. Through these rigorous tests in diverse populations, clinical trials not only validate the effectiveness of new interventions but also uncover potential side effects and long-term impacts. Ultimately, the impact of clinical trials extends beyond individual patients, shaping the landscape of healthcare and improving outcomes on a global scale [57]. However, while clinical trials are indispensable for advancing medical research and establishing the safety and efficacy of new interventions, they come with inherent limitations that can impact their efficiency, generalizability, and ability to address certain complex healthcare challenges. The limited patient diversity, stringent inclusion criteria, short follow-up durations, and ethical concerns are some of the limitations that pose obstacles to the comprehensive understanding of treatments and their real-world implications [58].

In recent years, the incorporation of AI technologies has become a transformative force, providing innovative solutions to address these challenges. This intersection of clinical trials and AI holds tremendous promise, providing tools and methodologies to enhance patient recruitment, optimize trial designs, streamline data management, and address ethical considerations [59,60,61,62].

Artificial intelligence has increasingly demonstrated its utility in enhancing the efficiency and precision of clinical trial processes, particularly in patient recruitment, trial design, and adaptive protocols. In the recruitment phase, AI models can analyze vast datasets—such as electronic health records, genomic data, and clinical notes—to identify individuals who match specific inclusion and exclusion criteria. This targeted approach not only accelerates recruitment but also improves population diversity and trial representativeness [63].

In trial design, AI-powered analytics enable the simulation of multiple study scenarios by integrating historical clinical data, disease progression models, and demographic variability. This allows researchers to optimize key parameters such as sample size, endpoint selection, and stratification strategies. Furthermore, adaptive trial protocols have benefited from real-time AI monitoring systems capable of analyzing incoming data streams during the trial. These systems support dynamic adjustments—such as dose modification, cohort expansion, or early termination—based on predefined statistical thresholds. By doing so, AI enhances both the ethical and scientific rigor of trials, minimizing patient exposure to ineffective interventions while maximizing data relevance and trial success rates [10,59,64].

In fact, a recent study analyzed the success of AI-discovered drugs in clinical trials, highlighting their growing impact on pharmaceutical research. The study found that AI-driven biotech companies have introduced 75 drug candidates into clinical trials since 2015, with 67 still progressing as of 2023. Notably, AI-discovered molecules demonstrated an 80–90% success rate in Phase 1 trials, significantly surpassing traditional drug development benchmarks. In Phase 2 trials, these drugs exhibited a 40% success rate, aligning with historical industry averages. The study categorized AI-discovered drugs into key areas, including small molecules, biologics, vaccines, and repurposed drugs, with oncology being a primary focus. Additionally, the rapid expansion of AI in drug discovery has accelerated development timelines and fostered collaborations between biotech startups and major pharmaceutical companies [65].

AI algorithms can analyze vast amounts of patient data from electronic healthcare records, the medical literature, and other sources to identify suitable candidates for clinical trials. Natural language processing (NPL) techniques enable the extraction of relevant information from unstructured data, helping researchers find individuals who meet specific criteria quickly and accurately [66].

Designing an effective clinical trial protocol is crucial for its success. AI-powered predictive analytics can analyze historical trial data to identify patterns, predict potential outcomes, and optimize study protocols. By considering various factors, such as patient demographics, disease characteristics, treatment protocols, and endpoints, AI algorithms can suggest adjustments to enhance trial effectiveness and efficiency [59,60]. In addition, AI algorithms can analyze patient data to predict treatment responses and outcomes. By integrating data from multiple sources, including genomics, proteomics, and clinical parameters, AI can identify biomarkers and patient subgroups that are more likely to respond favorably to a particular treatment. This predictive capability helps in personalizing treatment regimens and improving patient stratification in clinical trials [67].

AI-powdered monitoring systems can continuously analyze incoming data from clinical trials in real time. By detecting deviations from expected patterns and identifying safety concerns or efficacy signals early, AI enables researchers to make timely adjustments to trial protocols. This capability is particularly valuable in adaptative trials, where trial parameters can be modified dynamically based on accumulating data.

AI algorithms can assess patient risk factors and predict adverse events, allowing researchers to proactively mitigate risks and ensure patient safety during clinical trials. By analyzing patient characteristics, medical history, and treatment-related factors, AI can identify individuals who may be at higher risk of experiencing adverse events or treatment failures, enabling tailored interventions and monitoring strategies [68,69,70].

One of the most significant bottlenecks in clinical trials is patient recruitment. In fact, finding eligible patients who meet specific criteria can delay trials for months or even years, which drives up costs and postpones potentially life-saving treatments from reaching the public. AI addresses this challenge by analyzing vast datasets, such as electronic health records (EHRs), to identify potential participants based on factors like age, medical history, and genetic makeup. Using AI-powered tools, researchers can quickly match patients to trials, improving the chances of filling participant quotas efficiently. Furthermore, AI systems enhance retention rates by offering personalized outreach, sending tailored reminders, and providing ongoing support. These platforms keep participants engaged, reducing the risk of dropout and ensuring that trials can continue without disruptions.

To optimize patient selection for osteoarthritis (OA) clinical trials, a machine learning (ML) strategy was developed to identify individuals with a higher likelihood of disease progression. This “progression-enriched” approach aims to better evaluate the effectiveness of disease-modifying treatments by focusing on patients most likely to exhibit OA progression. The recruitment strategy was structured in two stages. In the first stage, ML models were trained on data from existing OA cohorts to rank candidates by their probability of progression. This model narrowed down the pool of potential participants for an initial screening visit. In the second stage, a refined ML model incorporated data gathered during screening to make the final patient selection. The effectiveness of this strategy was validated in the IMI-APPROACH knee OA study. Of 3500 candidates, 433 patients underwent screening, 297 were enrolled, and 247 completed a 24-month follow-up. The ML model demonstrated high accuracy in predicting pain-related progression (AUC 0.61). The progression rates in the selected population varied, with 30% showing pain-related progression, 13% showing structural changes, and 5% showing both. Furthermore, progressors were ranked higher than non-progressors, indicating the model’s success in identifying those more likely to experience disease progression. This ML-assisted recruitment strategy effectively enriched the trial population with progressive OA patients, demonstrating its potential to enhance trial efficacy. However, further refinement is needed to improve predictions for structural progression, and additional research is necessary to validate this approach in an interventional setting [71].

**Figure 6:**
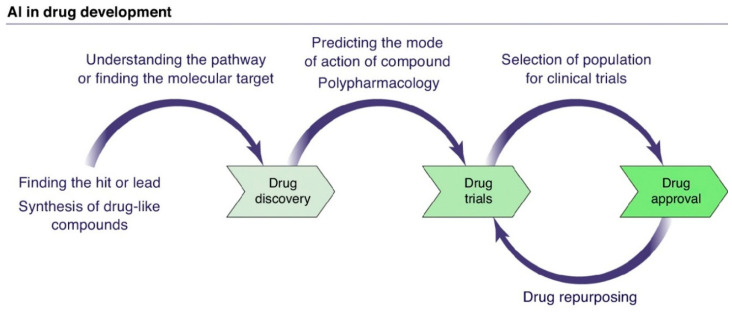
Schematic representation of AI application in drug development. Adapted with permission from Ref. [12]. Copyright 2019 Elsevier.

Figure 6 schematically presents the application of AI in drug development, illustrating the interaction between the different stages mentioned earlier.

Beyond structured clinical trial data, the integration of real-world data (RWD) and natural language processing (NLP) has significantly broadened the analytical capabilities of AI in pharmaceutical research. RWD encompass information collected from a variety of sources outside traditional clinical trials, such as electronic health records (EHRs), claims and billing data, mobile health apps, wearable sensors, patient registries, and even social media platforms. These data offer insights into patient behavior, adherence, comorbidities, and long-term treatment outcomes, providing a more comprehensive view of real-world clinical practice [72,73]. AI algorithms can process these large, heterogeneous datasets to identify patient subpopulations with specific clinical characteristics, predict progression trajectories, and simulate trial outcomes under various scenarios. For instance, machine learning models trained on EHR data can detect hidden phenotypes or patterns indicative of eligibility, thus enhancing patient stratification and reducing recruitment time. Moreover, RWD supports external control arms, allowing researchers to compare intervention groups with historical real-world cohorts, which is particularly valuable in rare diseases or ethical situations where placebo use is limited [74,75,76].

Complementing this, natural language processing (NLP) enables the extraction of structured information from vast amounts of unstructured text, such as clinical notes, pathology reports, radiology descriptions, and biomedical literature. NLP has been used to automatically identify adverse drug reactions, patient-reported outcomes, and even subtle clinical indicators not captured in standard coding systems. By integrating NLP outputs into AI models, researchers can uncover associations and patient characteristics that would otherwise remain inaccessible, enriching trial data and supporting adaptive protocols. Furthermore, NLP facilitates the automated screening of trial eligibility by parsing narrative health records, thereby streamlining recruitment workflows [77,78].

Artificial intelligence methods have also shown remarkable promise in the area of adverse drug reaction (ADR) detection, a critical component of post-marketing surveillance. A recent study introduced a novel deep learning framework known as Neural Self-Controlled Case Series (NSCCS), designed specifically for ADR discovery using large-scale electronic health records (EHRs). By following a self-controlled case series methodology, the model implicitly adjusts for time-invariant confounders, enhancing the reliability of the detected associations between drugs and adverse conditions. The framework was validated on real-world clinical data and demonstrated superior performance in identifying ADRs compared to traditional methods, illustrating the potential of deep neural networks to extract clinically actionable insights from complex longitudinal datasets [75]. Together, the synergistic use of RWD and NLP enhances the relevance, inclusiveness, and efficiency of clinical trials, bridging the gap between controlled experimental settings and real-world clinical environments.

**Figure 7:**
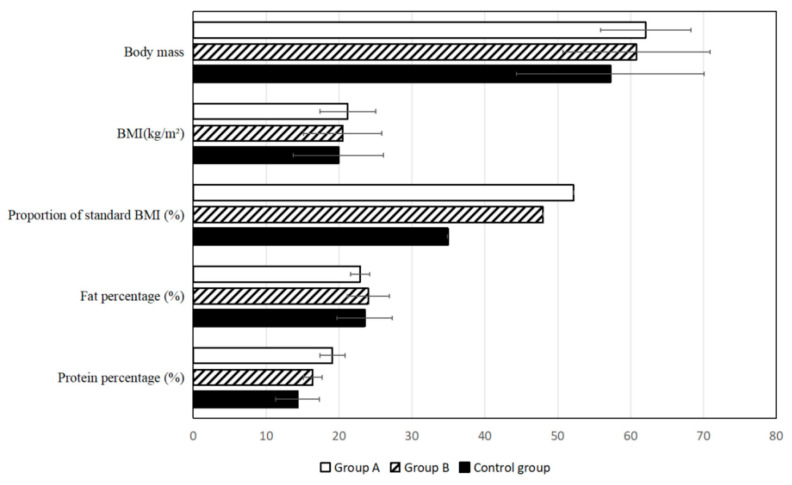
Comparative analysis of basic body quality indicators, including body mass index (BMI) and body protein levels, among patients with stage III–IV nasopharyngeal carcinoma (NPC) three years after treatment. Patients receiving AI-assisted home enteral nutrition (HEN) management (Group A) exhibited significantly better nutritional status than those under traditional HEN (Group B) or no HEN (control group) [79].

**Figure 8:**
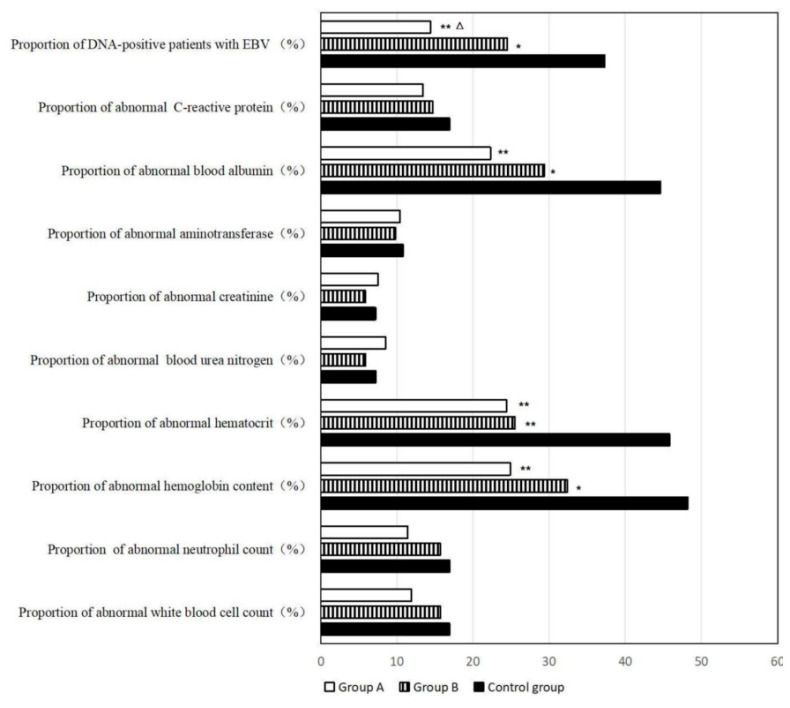
Laboratory results assessing hematologic and biochemical parameters among NPC patients three years post-treatment. AI-assisted HEN (Group A) was associated with higher hemoglobin and albumin levels and a lower Epstein–Barr virus (EBV) DNA positivity rate compared to the control group. * Compared with control group, 0.01 < *p* < 0.05; ** compared with control group, *p* < 0.01; △ compared with Group B, 0.01 < *p* < 0.05 [79].

Recent studies also demonstrate the potential of AI tools beyond trial design and recruitment, particularly in the context of post-treatment patient management and supportive care. For instance, a three-year clinical follow-up study in patients with stage III–IV nasopharyngeal carcinoma (NPC) showed that AI-assisted home enteral nutrition (HEN) management significantly improved long-term health outcomes. Patients who received AI-guided nutritional support exhibited higher survival rates, lower recurrence and metastasis, better nutritional profiles, and reduced psychological stress and physical discomfort when compared to standard care. These effects were clearly illustrated through objective physical and biochemical indicators, including body mass composition (Figure 7), and improved laboratory values, such as albumin and hemoglobin levels (Figure 8). These findings reinforce the relevance of AI not only in accelerating drug development but also in enhancing quality of life and treatment recovery trajectories [79].

### 3.3. AI-Developed Drugs: Highlighted Cases

Computational methods have played a crucial role in drug discovery. The use of medication design with the aid of computers has contributed to the development of several drugs that have already been approved, such as the initial examples of captopril, saquinavir, ritonavir, indinavir, and tirofiban [80].

In January 2020, the British pharmaceutical company Exscientia published that DSP-1181, a potent long-lasting 5-HT1A serotonin receptor agonist developed to treat OCD (Obsessive–Compulsive Disorder), had entered Phase 1 clinical trials. An AI platform was created to identify relevant compounds from chemical libraries. In partnership with the Japanese pharmaceutical company Sumitomo Dainippon Pharma, the drug took just 12 months to develop, from screening to the end of preclinical trials [81,82]. Exscientia has developed other drugs using AI, including DSP-0038, which entered Phase 1 clinical trials in the UK in May 2021. It was also developed in collaboration with Sumitomo Dainippon Pharma and aims to alleviate the behavioral and psychological symptoms of dementia, such as agitation, anxiety, and aggression, in individuals suffering from Alzheimer’s disease, as it has a low affinity for dopamine D2 receptors, improving its tolerability compared to conventional antipsychotics. EXS-21546, developed in December 2020, also entered Phase 1 clinical trials and acts as an immuno-oncology agent for various types of tumors. It was created in collaboration with Evotec [83].

Another example is Baricitinib, which was defined by BenevolentAI, a company that uses AI to discover new uses for existing drugs and to predict their effectiveness in treating SARS-CoV-2. It was found to be effective in reducing the virus’s ability to infect lung cells due to its anti-inflammatory and antiviral properties [84].

It is also relevant to mention a study that employed AI to screen 7684 molecules, training a predictive model to identify potential antibacterial compounds. The model then analyzed 6680 additional molecules and, within hours, successfully pinpointed 240 candidates for experimental testing. Among them, Abaucin emerged as a potent and highly selective antibiotic against *Acinetobacter baumannii*, a dangerous pathogen responsible for hospital-acquired infections. Unlike broad-spectrum antibiotics, Abaucin specifically targets *A. baumannii* by disrupting lipoprotein transport via the LolE protein, minimizing off-target effects on beneficial bacteria. In animal models, Abaucin demonstrated strong antibacterial activity, significantly reducing infection and inflammation in mice with *A. baumannii*-infected wounds. This breakthrough illustrates AI’s potential to accelerate drug discovery, optimize target specificity, and improve treatment efficacy, marking a shift toward more efficient and precise antibiotic development [85].

Another notable example is GENTRL, an AI-driven generative reinforcement learning model that successfully designed DDR1 kinase inhibitors, a promising drug class for treating fibrosis and cancer. Similarly, DeepMalaria, a deep learning-based model, identified compounds with Plasmodium falciparum inhibitory activity, contributing to malaria drug discovery. Another breakthrough is ReLeaSE, a reinforcement learning model capable of generating new drug-like molecules while predicting their biological activity with high accuracy. Additionally, AlphaFold, an AI-powered protein structure prediction tool, has transformed drug target identification by accurately predicting 3D protein structures, significantly improving drug design strategies. These advancements highlight AI’s ability to streamline drug development, optimize molecular properties, and repurpose existing drugs for new therapeutic applications, paving the way for more efficient and targeted treatments [86].

INS018_055 is an innovative drug candidate for idiopathic pulmonary fibrosis (IPF), a severe lung disease with limited treatment options. Developed by Insilico Medicine, this small molecule was designed using deep learning and generative AI models, which screened millions of chemical structures to predict an optimal candidate. AI significantly accelerated the drug’s development, allowing it to progress from discovery to Phase 1 clinical trials in record time. INS018_055 represents a breakthrough in AI-driven drug discovery, showcasing how machine learning can rapidly identify novel treatments for complex diseases [87].

Halicin is an antibiotic discovered through AI by a team at MIT. It was identified by a deep learning model known as Chem net, which was trained to predict antimicrobial activity across various compounds. The AI system searched chemical databases to find molecules with the potential to combat drug-resistant bacteria, and Halicin emerged as a promising candidate. Unlike conventional antibiotics, Halicin operates by disrupting the proton gradient across bacterial cell membranes, a mechanism that was previously unrecognized. The drug demonstrated broad-spectrum antimicrobial activity, effectively targeting a wide range of bacteria, including those resistant to multiple existing antibiotics. In preclinical tests, Halicin was shown to be effective against pathogens such as *Clostridium difficile*, *E. coli*, and *Mycobacterium tuberculosis*. Halicin represents a major breakthrough in the fight against antibiotic resistance, showcasing the potential of AI to accelerate drug discovery and provide novel solutions to longstanding global health challenges. Although still in the early stages of development, Halicin holds significant promise for tackling the growing threat of antibiotic-resistant infection [88].

Several artificial intelligence platforms have already demonstrated a concrete impact on pharmaceutical innovation, providing real-world examples of how these technologies are reshaping traditional drug discovery paradigms [36]. One of the most prominent is AlphaFold, developed by DeepMind, which revolutionized protein structure prediction. This platform employs deep learning techniques to accurately predict the three-dimensional structure of proteins based solely on their amino acid sequences, overcoming one of the major bottlenecks in structural biology. The wide accessibility of AlphaFold’s predictions has significantly accelerated the identification of novel drug targets, facilitating structure-based drug design [89,90]. Another noteworthy example is BenevolentAI, a platform that integrates machine learning with biomedical data mining to identify new therapeutic applications for existing drugs. During the COVID-19 pandemic, BenevolentAI successfully identified Baricitinib—a drug originally approved for rheumatoid arthritis—as a potential treatment for SARS-CoV-2 infection. The repurposed use of Baricitinib was rapidly validated through clinical trials and received emergency use authorization, demonstrating the practical relevance and speed that AI-based approaches can bring to drug development [91,92].

Atomwise is also at the forefront of AI in drug discovery. Using deep convolutional neural networks, their AtomNet platform performs structure-based virtual screening to predict binding affinities between small molecules and biological targets. This technology has been applied across multiple therapeutic areas, including oncology, neurology, and infectious diseases. Through partnerships with academic institutions and pharmaceutical companies, Atomwise has contributed to the identification of new candidate molecules, significantly reducing the time and resources required for early-stage discovery [93,94].

These platforms illustrate how AI can produce clinically meaningful results and support the development of innovative therapies with enhanced precision and efficiency.

### 3.4. AI in Biomedical Systems Modeling and Network Dynamics

In complex biomedical and pharmacological systems, where feedback loops and time-delayed interactions are common, identifying the underlying network structure in a timely manner is crucial for accurate modeling and intervention. While traditional topology identification techniques often rely on asymptotic convergence, recent advances in finite-time topology identification offer faster and more robust alternatives. A novel method based on finite-time stability theory has demonstrated effective synchronization and topology reconstruction of delayed dynamical networks, with potential implications for biological systems where dynamic connectivity influences drug response. Although initially applied to power grids for rapid detection of line outages, the proposed method proved capable of fast and accurate topology identification, even in second-order nonlinear systems. Future developments—including the incorporation of switching topologies and resilience against cyber attacks—may further enhance its applicability in healthcare-related network modeling [95].

### 3.5. Limitations, Risks, and Real-World Performance of AI Applications

Despite the growing number of AI-driven tools in pharmaceutical R&D, several critical limitations continue to hinder their effective translation into real-world success. One of the major challenges is data heterogeneity. Pharmaceutical data originate from highly diverse sources, including omics datasets, clinical records, imaging, and chemical structures, which often differ in format, quality, scale, and completeness. These discrepancies can impair model performance, reduce reproducibility, and require extensive preprocessing and standardization [96].

Another major concern is algorithmic bias, which arises when training datasets are unbalanced or not representative of the target population. This can lead to biased predictions, poor generalizability, and potential safety risks, especially when AI models are applied to underrepresented groups in clinical settings [96,97]. Model interpretability also remains a significant barrier. Many deep learning approaches operate as “black-box” systems, making it difficult for researchers and regulators to understand the rationale behind specific predictions. The emergence of explainable AI (XAI) seeks to mitigate this by providing interpretable outputs and highlighting key variables that influence model decisions. However, implementation in regulated environments still faces resistance due to the complexity of the models [91,96,97]. Equally important is the lack of real-world validation for many AI-derived insights. While models may perform well in retrospective or simulated datasets, prospective validation in live clinical or regulatory contexts is rare. This limits confidence in AI-generated drug candidates and slows adoption.

Although there have been notable successes, such as *Baricitinib*—repurposed for COVID-19 treatment using AI-based target prediction—and the rapid design of *DSP-1181*, failures are less often reported. Several AI-predicted candidates have shown promise in silico but failed to replicate efficacy in vitro or in early clinical phases, often due to overfitting, flawed assumptions, or lack of biological plausibility [91,92]. For AI to become a dependable tool in drug development, robust data governance, external validation, and transparent reporting standards must be established, ensuring that AI models are not only innovative but also reproducible, equitable, and clinically meaningful.

## 4. Regulatory Issues

The integration of AI and ML into pharmaceutical research and development presents significant regulatory challenges. Existing regulatory frameworks were not initially designed to evaluate AI-driven therapeutics, and their rapid evolution has outpaced the development of specific guidelines tailored to these tools [98,99]. However, regulatory agencies worldwide are actively adapting their frameworks to the unique challenges posed by AI and ML in pharmaceutical research and healthcare. For example, the U.S. Food and Drug Administration (FDA) has launched targeted programs, such as the Digital Health Software Precertification (Pre-Cert) Program and the Artificial Intelligence/Machine Learning-Based Software as a Medical Device (SaMD) Action Plan. These initiatives aim to streamline the evaluation and approval of AI-driven tools while ensuring patient safety and efficacy [100,101]. Notably, AI-based medical devices like IDx-DR, an autonomous system for diabetic retinopathy diagnosis, have already received FDA clearance, exemplifying the agency’s proactive stance toward validated AI technologies [101].

Recent regulatory reviews emphasize the growing consensus on the need for risk-based oversight frameworks to ensure the ethical, safe, and scalable use of AI in drug and biological product development. A multidisciplinary review reflecting on FDA workshops has highlighted the importance of practical regulatory guidelines, cross-border harmonization, and the integration of human oversight in AI-assisted decision-making processes. These reflections underscore that beyond technical validation, regulatory approaches must also address broader issues such as data privacy, transparency, governance, and equitable access. As global agencies navigate differing regulatory perspectives, a unified ethical and legal framework for AI in drug development is becoming increasingly vital [97].

One of the central concerns lies in the lack of clear, standardized guidance on how to evaluate and validate AI-derived predictions and computational models. Unlike conventionally developed molecules—typically assessed through well-established experimental paradigms—AI-generated candidates often arise from complex, opaque algorithms, particularly deep learning models, which may lack interpretability and mechanistic transparency. This “black-box” nature can hinder regulatory confidence and pose challenges to the assessment of safety, efficacy, and reproducibility. To address these issues, agencies such as the FDA have introduced several initiatives. For instance, the FDA’s “Artificial Intelligence/Machine Learning (AI/ML)-Based Software as a Medical Device (SaMD) Action Plan” emphasizes the importance of flexibility in oversight while maintaining rigorous standards of patient safety. A key element of this initiative is the development of “Predetermined Change Control Plans” (PCCPs), which allow for software updates without resubmission, provided that modifications are pre-specified and controlled. Complementing this, the FDA is developing Good Machine Learning Practice (GMLP) guidelines, in collaboration with organizations like IEEE and ISO, to ensure algorithmic quality, transparency, and reliability [98,100].

Additionally, the FDA has launched the Digital Health Software Precertification (Pre-Cert) Program and the Emerging Technology Program to accelerate the evaluation and approval of novel AI-based tools [101]. Real-world regulatory approvals, such as the clearance of IDx-DR—an autonomous AI diagnostic system for diabetic retinopathy—demonstrate the agency’s openness to validated AI applications. Similarly, the FDA’s Real-World Performance (RWP) Monitoring program seeks to track the post-market performance of AI/ML-based tools to ensure ongoing safety and efficacy [102].

In Europe, the EMA has taken similar steps through its Innovation Task Force, which provides early dialogue with developers of AI-driven health technologies. The EMA has stressed the importance of transparency, robust data quality, and post-market surveillance in submissions that involve AI components. Under the EU’s Medical Device Regulation (MDR), AI-based medical devices must undergo stringent classification and conformity assessments. However, both the FDA and EMA continue to face challenges in regulating continuously learning AI systems, particularly with respect to algorithmic drift and the need for dynamic risk assessment [103].

The successful integration of physiologically based pharmacokinetic (PBPK) models into regulatory submissions provides a relevant precedent for the future acceptance of AI tools. Originally limited to exploratory simulations, PBPK models are now routinely used to inform dosing strategies, predict drug–drug interactions, and guide extrapolations to special populations. Their journey to regulatory acceptance—through systematic validation, standardization, and inclusion in guidance documents—highlights a viable pathway for future AI technologies [104].

Another emerging regulatory frontier is the incorporation of in silico clinical trials and virtual patient populations. These AI-driven simulations, powered by real-world data and mechanistic models, allow researchers to predict drug responses, optimize protocols, and explore therapeutic scenarios without direct patient involvement. Although not a replacement for traditional clinical trials, they are increasingly viewed as valuable complements that can enhance trial design and improve resource efficiency [105,106]. At the same time, several risks require careful consideration. Data quality and bias represent significant regulatory hurdles, as training data may be unbalanced or non-representative, potentially leading to discriminatory outcomes. As such, regulators are emphasizing the need for fairness audits, diverse training datasets, and post-market surveillance to detect algorithmic biases [107].

The issue of interpretability is also central. Regulatory bodies require explainable AI (XAI) approaches that can justify molecular selection and decision-making processes, especially in contexts involving patient safety. Companies are responding by incorporating XAI methods into development pipelines, aiming to build models that are both powerful and interpretable [108]. Another complex challenge is the question of intellectual property (IP) rights for AI-generated drug candidates. Current patent systems were designed around human inventorship, and legal frameworks are still evolving to determine how ownership is assigned when AI plays a central role in molecular design. These raise questions about attribution, responsibility, and protection of innovation.

Finally, the lack of harmonized global standards remains a barrier. Regional disparities in classification, approval, and surveillance practices can hinder the efficient development and deployment of AI-enabled pharmaceutical products. Collaborative initiatives and international regulatory convergence will be essential to foster a balanced, globally coherent approach [96].

In summary, the regulatory landscape for AI in drug discovery is rapidly evolving. While substantial challenges persist—such as interpretability, bias, and lack of harmonization—regulatory bodies are increasingly proactive in addressing these through flexible oversight models, stakeholder collaboration, and adaptive guidance frameworks. Continued dialogue between developers, regulators, and academia will be key to ensuring that AI technologies are safely and effectively integrated into the pharmaceutical innovation pipeline.

## 5. Conclusions and Future Perspectives

These investments are forming a clear vision that AI will play a significant role in the future discovery and development of drugs. The fast advancement of AI (AI) is contributing to innovation across numerous industries, with the pharmaceutical sector being one of the most impacted. AI aims to automate, optimize, and personalize various areas within the pharmaceutical industry, particularly in pharmacological research, which shows the most interest among researchers [28,29].

Virtual screening techniques will help accelerate the drug discovery process by analyzing vast chemical libraries and finding the best therapeutic candidate based on the required characteristics. This process will significantly reduce the time spent discovering potential therapeutic compounds and also cut down on associated costs [13].

Patient categorization through AI will facilitate the development of personalized therapies by analyzing genomes, proteomes, and clinical records. This will allow for the creation of a more effective medication for each specific patient, with fewer adverse reactions and a tailored dose. With AI algorithms, it will be possible to improve therapeutic outcomes by considering factors such as age, weight, genetics, and disease status. Using devices to collect real-time data from patients will enable the proposal of personalized therapies, helping in the development of suitable and effective medications [13].

Clinical trials recruit volunteer patients to aid in the drug development process. For the results to be as reliable as possible, a patient selection process must be in place. AI will allow access to records of biomarkers, genetic profiles, and patient health status, enabling the identification of suitable patients, thus reducing costs and speeding up the process [13].

However, the continued use of AI in drug formulation raises ethical and regulatory concerns that must be carefully examined. Key issues include the transparency of algorithms, the protection of patient personal data, and ensuring that technologies are used in a fair and safe manner.

As AI evolves and integrates with the pharmaceutical sector, it is crucial that the industry and regulatory bodies work together to harness this technology and minimize potential risks, ensuring that everyone benefits from the proposed innovations in a fair and safe way.

## Figures and Tables

**Table 2:** Ethical principles of AI in health identified by the WHO [14,21].

Ethical Principle	Ethical Foundations
**Autonomy**	- The use of machines must not undermine human autonomy. - Design AI systems that help humans make informed decisions. - Humans can decide whether to use an AI system to reach a final decision.
**Public interest**	- Ensure safety, accuracy, and effectiveness requirements are met before implementing any AI technology. - Measures must ensure ongoing control and improvement. - Monitor AI algorithm performance to prevent patients or groups from suffering mental or physical harm. - Protect individuals from any form of stigmatization or discrimination due to their health status.
**Transparency**	- Implementation of documents with sufficient information for AI technology implementation. - Improve system quality and protect patients and public health. - Identify errors. - Adequate and effective supervision. - Include accurate information on the limitations and assumptions of the technology, algorithm development, operational protocols, and data properties. - Educational information adapted to the population.
**Responsibility**	- Minimize or avoid errors. - Use of technology in a responsible manner. - Identify all parties involved in the development and implementation of the technology and assign collective responsibilities. - Support justice in resolving issues.
**Inclusion and equity**	- Hiring staff from diverse backgrounds, cultures, and disciplines. - Design and evaluation of technologies by those who will be directly affected by them. - Adoption of open-source software available in both high and low-income countries. - Diversity in languages and communication methods. - Unbiased technologies.
**Privacy**	- Protect the confidentiality and privacy of individuals and ensure informed consent.

**Table 3:** Examples of drugs subject to the mechanism of repurposing using AI techniques [47].

Molecule	Original Therapeutic Indication	New Therapeutic Indication
Ormeloxifene	Oral contraceptive	Prostate Cancer
Metformin	Diabetes	Cancer
Cimetidine	Ulcer	Lung Cancer
Bazedoxifene	Osteoporosis	Breast Cancer

## Data Availability

No new data were created or analyzed in this study. Data sharing is not applicable to this article.

## Abbreviations

ADME	Absorption, Distribution, Metabolism, Excretion
ANN	Artificial Neural Network
DL	Deep Learning
DNNs	Deep Neural Networks
DTBA	Drug–Target Binding Affinity
EPI	Estimation Program Interface
FDA	Food and Drug Administration
R&D	Research and Development
AI	Artificial Intelligence
ML	Machine Learning
MMP	Matched Molecular Pair
NN	Neural Forest
QSPR	Quantitative Structure–Property Relationship
SaMD	Software as a Medical Device
SMILE	Simplified Molecular Input Line Entry System
SVM	Support Vector Machine
WHO	World Health Organization
